# Longitudinal studies that use data collected as part of usual care risk reporting biased results: a systematic review

**DOI:** 10.1186/s12874-017-0418-1

**Published:** 2017-09-06

**Authors:** Delaram Farzanfar, Asmaa Abumuamar, Jayoon Kim, Emily Sirotich, Yue Wang, Eleanor Pullenayegum

**Affiliations:** 1 University Health Network, University of Toronto, Toronto, M5T 2S8 Canada; 2 Institute of Medical Science, University of Toronto, City, ON M5S 1A8 Canada; 3 Faculty of Arts & Science, University of Toronto, City, ON M5S 3G3 Canada; 4 Child Health Evaluative Sciences, The Hospital for Sick Children, Toronto, ON M5G 1X8 Canada; 5 Dalla Lana School of Public Health, University of Toronto, City, ON M5T 3M7 Canada

**Keywords:** Longitudinal data, Administrative data, Statistical methods, Bias

## Abstract

**Background:**

Longitudinal studies using data collected as part of usual care risk providing biased results if visit times are related to the outcome of interest. Statistical methods for mitigating this bias are available but rarely used. This lack of use could be attributed to a lack of need or to a lack of awareness of the issue.

**Methods:**

We performed a systematic review of longitudinal studies that used data collected as part of patients’ usual care and were published in MEDLINE or EMBASE databases between January 2005 through May 13^th^ 2015. We asked whether the extent of and reasons for variability in visit times were reported on, and in cases where there was a need to account for informativeness of visit times, whether an appropriate method was used.

**Results:**

Of 44 eligible articles, 57% (*n* = 25) reported on the total follow-up time, 7% (*n* = 3) on the gaps between visits, and 57% (*n* = 25) on the number of visits per patient; 78% (*n* = 34) reported on at least one of these. Two studies assessed predictors of visit times, and 86% of studies did not report enough information to assess whether there was a need to account for informative follow-up. Only one study used a method designed to account for informative visit times.

**Conclusions:**

The low proportion of studies reporting on whether there were important predictors of visit times suggests that researchers are unaware of the potential for bias when data is collected as part of usual care and visit times are irregular. Guidance on the potential for bias and on the reporting of longitudinal studies subject to irregular follow-up is needed.

**Electronic supplementary material:**

The online version of this article (10.1186/s12874-017-0418-1) contains supplementary material, which is available to authorized users.

## Background

Longitudinal studies are vital to understanding disease progression. Chart reviews are a common source of longitudinal data, and can be used to identify the long-term benefits of a medical intervention, risk factors for poor outcomes, and the burden of disease over time. Chart reviews are inexpensive and popular; for example, they are estimated to comprise 25% of all scientific articles published in emergency medicine journals [1]. However, chart reviews often feature irregular follow-up times, i.e. visit times that vary among patients, often to the extent that no two patients share an observation time. If patients visit more often when unwell, this can lead to a biased picture of disease course unless the data are analyzed appropriately [2].

Many analyses of longitudinal data subject to irregular observation use traditional approaches to longitudinal data analysis such as generalized estimating equations (GEEs) [3] and linear mixed models [4]. While these methods can be run on data with irregular follow-up, they will give biased inferences if the visit intensity is related to the outcome [5]. For this reason, methods designed specifically for irregular observation are usually required.

Statistical methods to handle longitudinal data subject to irregular follow-up began to be developed in the 1990s [6, 7]. There is now a substantial literature on these methods, which include inverse-intensity weighting [2, 8–10] and semiparametric joint models [11–14]. Although specifically developed to help medical researchers by addressing the problem of over-representation of certain individuals or certain types of measurements in longitudinal studies with irregular follow-up, their use remains limited. A 2015 citation analysis using the Web of Science revealed that these methods were used only once as the primary analysis [15] and applied twice as a sensitivity analysis [16, 17].

These methods are either not being used because they are not needed or because there is a knowledge translation gap. This paper aimed to assess whether the lack of use is due to a lack of need. Specifically, we used a systematic review to address the following questions: Among longitudinal studies published in the medical literature that used data collected as part of patients’ usual care, and that were published in the period January 2005 to May 2015, 1. what proportion reported summary statistics on a) the number of visits per patient, b) gaps between visits, c) total follow-up time; 2. was there an assessment of predictors of visit time, and if so, was there a need to account for the fact that visit time was irregular; 3. was a method used that accounted for potential informativeness of visit times? The first question addresses whether the extent of irregularity was reported, the second whether visit times were informative about the outcome, and the third whether an appropriate method was used.

## Methods

This review did not include outcomes of direct patient or clinical relevance and was thus not eligible for registration in Prospero (International Prospective Register of Ongoing Systematic Reviews, http://www.crd.york.ac.uk/prospero) [18, 19].

### Search

We performed a search of the MEDLINE and EMBASE databases to identify studies assessing longitudinal data collected as part of patients’ usual care (see Additional file 1 for search terms). For both databases, the earliest publication date was restricted to January 2005, since several methods for analyzing longitudinal data subject to irregular follow-up were proposed by this time [6, 7], and the latest publication date was May 13, 2015.

### Study selection and eligibility criteria

Eligibility criteria were chosen so as to specify studies where follow-up would be expected to be irregular, and where inverse-intensity weighting or semi-parametric joint modelling would be an appropriate method of analysis. Our analysis was limited to articles published in English.

We included studies that used patient-level data collected as part of patients’ usual care with an outcome that was measured on at least three occasions. We excluded studies that met one or more of the following criteria: 1) outcome was assessed on fewer than three occasions; 2) outcome was whether or not a visit occurred, or the number of visits; 3) visit times were specified by protocol, or analysis restricted to visits at specified times; 4) time-to-event analyses; 5) outcome was a single binary outcome per patient; 6) the outcome could have occurred only if a visit occurred; 7) outcome was measured on aggregate data. In addition, systematic reviews, meta-analysis and randomized controlled trials were also excluded.

We combined the searches from MEDLINE and EMBASE, removed duplicates and screened abstracts for eligibility. In the summer of 2016 (May–September) we trained a team of four reviewers (AA, JK, ES, YW) and two reviewers were chosen at random for each paper. These reviewers independently assessed both the abstracts and full-text articles, made eligibility decisions and resolved disagreements by discussion. If necessary, a third party was consulted. As our reviewers were working part time, not all papers were assessed during this time, and the remainder were assessed by DF and EP. The same template was provided to each reviewer to record their results. In the first stage, abstracts were classified as either ineligible based on the above inclusion and exclusion criteria, or as needing full-text review. In the second stage, the full-texts were reviewed for abstracts that were not excluded. Agreement between reviewers was assessed using Cohen’s kappa [20].

### Data extraction

The following data were extracted independently by two reviewers (DF and EP), with discrepancies resolved by consensus: descriptive data on the number of visits per patient (e.g. mean, median, range); descriptive data on gaps between visits; descriptive data on follow-up time (e.g. maximum follow-up time, median follow-up); how the longitudinal data was analyzed (methods used, covariance structure reported, rationale explained); whether participants were enrolled prospectively; whether there was a clearly defined end of the study, and if so, how many participants were followed to the end of the study; whether characteristics of those lost to follow-up were compared with those who were not; whether there was an assessment of predictors of visit times, and if so, how this was assessed (e.g. recurrent event regression); whether there was a need to account for the fact that visit time was irregular, and if so, whether the statistical analysis accounted for it. The statistical literature indicates that visit irregularity should be accounted for if it is informative, that is, if the visit and outcome processes are not independent. This could happen if there were a covariate (observed or unobserved) that was associated with both the outcome and the visit times. For example, if the outcome of interest is blood pressure and older patients tend to have higher blood pressure and also more measurements, then the visit scheme is informative. Thus if analysis of visit times uncovers a predictor that is also a predictor of outcome, the visit times are informative and should be accounted for. We distinguished between papers that reported results of analysis intended to assess whether the visit scheme was informative (i.e. an assessment of predictors of visit times, e.g. through recurrent event analysis of the visit process), papers where an informative visit scheme could be deduced based on other information in the paper (e.g., descriptive statistics on length of follow up or number of visits, separately for certain subgroups), and papers where it was not possible to tell whether the visit scheme was informative because insufficient analysis was reported.

Results were summarized using percentages.

### Assessment of study quality

The Newcastle-Ottawa Scale (NOS) [21] was used to assess the quality of included studies in this systematic review. Each study was evaluated based on the NOS scale for fulfilling the established criteria in NOS for the 3 components of selection, comparability and outcome. An overall quality score was calculated by adding the number of stars for each category for a maximum total of 9.

## Results

**Fig. 1:**
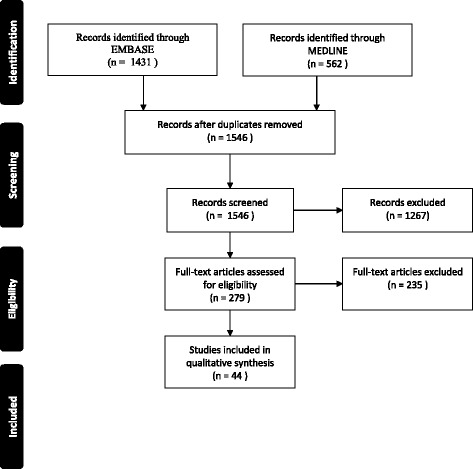
PRISMA flow diagram

The search identified 1546 articles, of which 279 proceeded to full-text review, and 44 were included in final analysis (See Fig. 1). The reviewers agreed in their inclusion/exclusion decision in 96% of the 1546 articles, with a kappa of 0.57. We found that the proportions of articles that reported summary statistics on the number of visits per patient, gaps between visits and the total follow-up time were 57% (*n* = 25), 7% (*n* = 3) and 57% (*n* = 25), respectively (Table 1). Twenty-two percent (*n* = 10) of articles did not provide summary statistics on any of the above (See Table 2).

**Table 1:** Summary statistics on reporting of visit irregularity, predictors of visit times, and methods of analysis

Outcomes of Interest	N (out of 44)	%
Study design		
Prospective	10	23
Retrospective	31	70
Unclear	3	7
Clearly defined end of study		
Yes	34	77
No	10	23
Comparison of those with and without full follow-up among studies with a clearly defined end of follow-up	(out of 34)	
Yes	5	15
No	24	71
Not Applicable (all participants had full follow-up)	5	15
Method of analysis		
Linear or logistic regression	8	18
Gaussian process regression	1	2
Repeated measures	11	25
Mixed model or generalized mixed model	20	45
GEE	3	7
IIW-GEE	1	2
Reported summary statistics on		
Number of visits per patient	25	57
Gaps between visits per patient	3	7
Follow-up time per patient	25	57
Predictors of visit time assessed		
Yes	2	5
No	41	93
Unclear	1	2
Was there a need to account for informative visit times?		
Yes	6	14
of which		
Analysis specifically designed to check for informativeness	1 (out of 6)	18
Informativeness inferred by reviewers	5 (out of 6)	82
Unclear	38	86
Method used to account for informative visit times for studies with sufficient reporting of an identifiable need	(out of 6)	
Yes	1	19
No	5	81

**Table 2:** Descriptive information and extracted variables of interest for included studies

ID	Study		Study Design	Sample Size		Eligible Study outcome	Country		Method of analysis
1	Adams, et al. (2008)		Retrospective	1806		Hemoglobin A1C levels	USA		Mixed model
2	Astrom, et al. (2014)		Unclear	339		Intraocular pressure change	Sweden		Mixed model
3	Bernstein, et al. (2005)		Retrospective	47		Mean arterial pressure	USA		Repeated measures
4	Biskupiak, et al. (2010)		Retrospective	47,796		Blood pressure goals	USA		Logistic regression
5	Bradford, et al. (2006)		Retrospective	50,741		Low-density lipoprotein goals	USA		Logistic regression
6	Cheung, et al. (2013)		Retrospective	94		DBS electrode impedance	USA		Mixed model
7	Coplan,et al. (2005)		Retrospective	91		Childhood Autism Rating Scale	USA		Mixed model
8	Dhawale, et al. (2013)		Retrospective	7		Peak inspiratory pressure	USA		Repeated measures
9	Elmelund, et al. (2014)		Retrospective	119		Plasma Creatinine levels	Denmark		Mixed model
10	Fattah, et al. (2014)		Retrospective	10		Cephalometric outcomes	Canada		Repeated measures
11	Fatti, et al. (2010)		Retrospective	2332		Virological suppression, weight	South Africa		GEE
12	Flack, et al. (2007)		Unclear	459		Blood pressure response	USA		Mixed model
13	Fong, et al. (2009)		Prospective	408		Cognitive decline	USA		Mixed model
14	Gao, et al. (2014)		Prospective	2906		Changes in Blood pressure	USA		Linear regression
15	Ghate, et al. (2013)		Retrospective	3038		Metabolic parameter monitoring	USA		Linear regression
16	Gofman, et al. (2009)		Retrospective	95		Development of obesity	USA		Mixed model
17	Guelinckx, et al. (2010)		Retrospective	605		Weight gain	Belgium		Mixed model
18	Haas, et al. (2012)		Retrospective	413		Weight loss	USA		Repeated measures
19	Heintzelman, et al. (2013)		Retrospective	33		Pain	Finland		Logistic regression
20	Henes, et al. (2010)		Retrospective	109		Eating and TV behavior	USA		Repeated measures
21	Jehi, et al. (2011)		Prospective	5960		Quality of life	USA		GEE
22	Kharbanda, et al. (2014)		Retrospective	510		Changes in BMI, blood pressure	USA		Mixed model
23	Lasko, et al. (2013)		Retrospective	4360		Unsupervised feature learning	USA		Gaussian regression
24	Maahs, et al. (2007)		Retrospective	360		Total cholesterol, HDL	USA		Mixed model
25	Mahmud, et al. (2010)		Prospective	190		Response to viral infection	Pakistan		Repeated measures
26	Mancevski, et al. (2007)		Retrospective	99		Schizophrenia symptoms	USA		Repeated measures
27	McCoy, et al. (2006)		Retrospective	41		Weight gain	USA		Mixed model
28	Nannetti, et al. (2009)		Prospective	395		Post-stroke recovery	Italy		Repeated measures
29	Pan, et al. (2010)		Prospective	253		Infant growth	USA		Mixed model
30	Patterson, et al. (2009)		Prospective	90		Pulmonary function, weight	USA		Mixed model
31	Pirraglia, et al. (2012)		Prospective	97		Blood pressure goals	USA		Repeated measures
32	Roth, et al. (2010)		Retrospective	102		Disease severity	Canada		Linear regression
33	Ruiz, et al. (2013)		Unclear	701		Mini Mental State Examination	Spain		Mixed model
34	Sarafoglou, et al. (2014)		Retrospective	104		Adult Height	USA		Mixed model
35	Schwartz, et al. (2014)		Retrospective	163,820		Body Mass Index trajectory	USA		Mixed model
36	Snijder, et al. (2012)		Prospective	4680		Fetal growth	Netherlands		Mixed model
37	Sy, et al. (2008)		Retrospective	58		Weight-for-age	Canada		Repeated measures
38	Tamayo, et al. (2015)		Retrospective	725		Obesity	Canada		GEE
39	Tanabe, et al. (2012)		Prospective	342		Changes in pain scores	USA		Linear regression
40	Ting, et al. (2005)		Retrospective	120		Intensity of treatment	USA		Linear regression
41	Ullrich, et al. (2013)		Retrospective	286		Pain and depression measures	USA		Repeated measures
42	Walker, et al. (2009)		Retrospective	119		Quality of life	USA		Mixed model
43	Wong, et al. (2012)		Retrospective	11,735		BMI trajectories	USA		IIW-GEE
44	Zechmann, et al. (2009)		Retrospective	39		Prostate gland volume	Germany		Mixed model
ID	Study	Number of visits provided	Gaps between visits provided	Total follow-up time provided	Assessment for predictors of visit times provided	Need a method that accounts for irregularity	Method to account for irregularity used	Clearly defined end of study	Comparison of those followed for duration of interest vs not
1	Adams, et al. (2008)	No	No	Yes	No	Unclear	No	Yes	No
2	Astrom, et al. (2014)	Yes	Yes	Yes	No	Unclear	No	Yes	No
3	Bernstein, et al. (2005)	No	No	Yes	No	Unclear	No	Yes	No
4	Biskupiak, et al. (2010)	No	No	Yes	No	Unclear	No	Yes	No
5	Bradford, et al. (2006)	No	No	No	No	Unclear	No	Yes	No
6	Cheung, et al. (2013)	Yes	No	No	No	Unclear	No	Yes	No
7	Coplan,et al. (2005)	Yes	No	Yes	No	Unclear	No	No	n/a
8	Dhawale, et al. (2013)	Yes	Yes	Yes	No	Unclear	No	No	No
9	Elmelund, et al. (2014)	No	No	No	No	Unclear	No	Yes	No
10	Fattah, et al. (2014)	Yes	No	Yes	No	Unclear	No	No	No
11	Fatti, et al. (2010)	No	No	Yes	No	Yes	No	Yes	Yes
12	Flack, et al. (2007)	Yes	No	Yes	No	Unclear	No	No	No
13	Fong, et al. (2009)	No	No	No	No	Unclear	No	Yes	No
14	Gao, et al. (2014)	No	No	Yes	No	Yes	No	Yes	Yes
15	Ghate, et al. (2013)	No	No	No	No	Unclear	No	Yes	No
16	Gofman, et al. (2009)	No	No	Yes	No	Unclear	No	No	Yes
17	Guelinckx, et al. (2010)	Yes	No	No	No	Unclear	No	Yes	n/a
18	Haas, et al. (2012)	No	No	No	No	Yes	No	Yes	No
19	Heintzelman, et al. (2013)	Yes	No	Yes	No	Unclear	No	Yes	n/a
20	Henes, et al. (2010)	Yes	No	No	No	Unclear	No	Yes	No
21	Jehi, et al. (2011)	Yes	No	No	No	Unclear	No	Yes	No
22	Kharbanda, et al. (2014)	No	No	No	No	Unclear	No	Yes	No
23	Lasko, et al. (2013)	No	No	No	No	Unclear	No	No	No
24	Maahs, et al. (2007)	Yes	No	Yes	No	Unclear	No	Yes	No
25	Mahmud, et al. (2010)	No	No	No	No	Unclear	No	Yes	No
26	Mancevski, et al. (2007)	No	No	Yes	No	Yes	No	Yes	n/a
27	McCoy, et al. (2006)	Yes	No	Yes	No	Unclear	No	No	No
28	Nannetti, et al. (2009)	Yes	No	Yes	No	Unclear	No	Yes	No
29	Pan, et al. (2010)	Yes	No	Yes	No	Unclear	No	Yes	No
30	Patterson, et al. (2009)	Yes	No	No	No	Unclear	No	Yes	No
31	Pirraglia, et al. (2012)	Yes	No	No	No	Unclear	No	Yes	No
32	Roth, et al. (2010)	No	No	Yes	No	Unclear	No	Yes	n/a
33	Ruiz, et al. (2013)	No	No	No	No	Unclear	No	No	No
34	Sarafoglou, et al. (2014)	No	No	Yes	No	Unclear	No	Yes	No
35	Schwartz, et al. (2014)	Yes	Yes	Yes	No	Unclear	No	Yes	Yes
36	Snijder, et al. (2012)	Yes	No	Yes	No	Unclear	No	Yes	No
37	Sy, et al. (2008)	No	No	No	No	Unclear	No	Yes	No
38	Tamayo, et al. (2015)	Yes	No	Yes	No	Unclear	No	Yes	No
39	Tanabe, et al. (2012)	Yes	No	No	No	Unclear	No	Yes	n/a
40	Ting, et al. (2005)	Yes	No	No	No	Unclear	No	Yes	No
41	Ullrich, et al. (2013)	Yes	No	Yes	Yes	Yes	No	Yes	Yes
42	Walker, et al. (2009)	Yes	No	No	No	Unclear	No	No	No
43	Wong, et al. (2012)	Yes	No	Yes	Yes	Yes	Yes	Yes	Yes
44	Zechmann, et al. (2009)	Yes	No	Yes	No	Unclear	No	No	No

The majority of articles (93%, *n* = 41) did not assess predictors of visit time. In 38 articles (86%), there was insufficient analysis to determine whether there was a need to account for informative visit times, and in the remaining 6 studies, this need was present. Only one of these 6 studies detailed analysis in the methods section that was intended to check for predictors of visit times (i.e. an informative visit scheme) [22] . In four of the 6 studies, the reviewers inferred that visit times were informative: one study provided results demonstrating that age was a predictor of visiting [23]; a further three studies reported predictors of the total length of follow-up [24–26]; and in the remaining study, it was known by design that high-risk patients were asked to visit more often [27].

Thirty-one of 44 articles (70%) used mixed models or repeated measures to analyze outcomes. In two cases data was reduced before using repeated measures (once by taking a mean within pregnancy trimesters, once by using the first three measurements only). Only one study used a method specifically designed to handle informative visit times, namely an inverse-intensity weighted GEE [2, 22] .

**Fig. 2:**
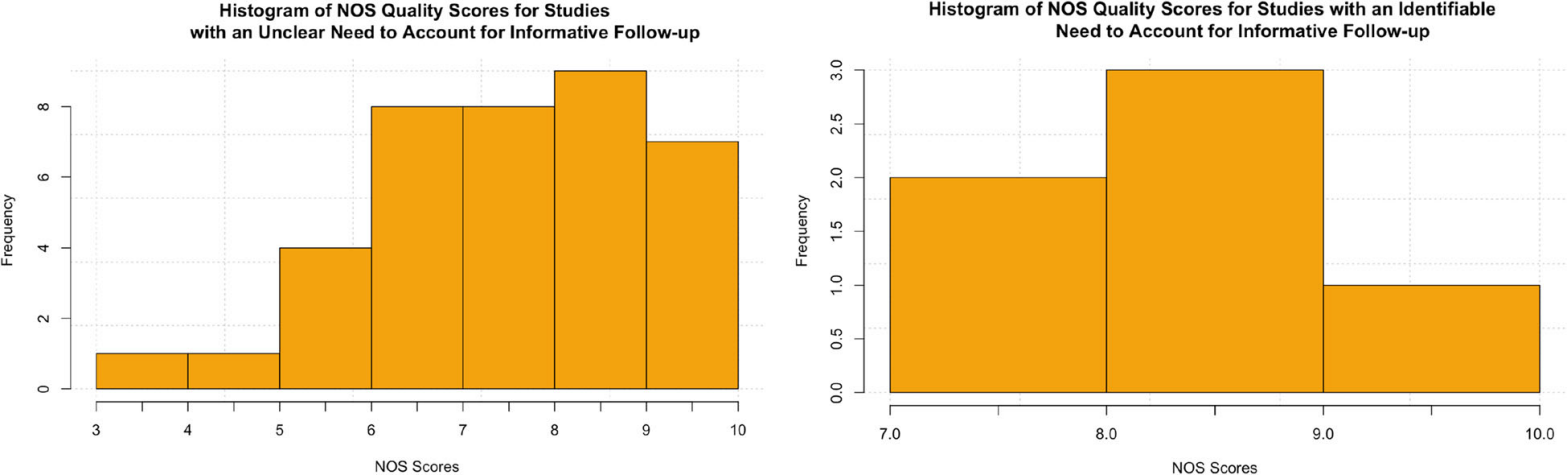
NOS Overall Quality Scores for included studies

The mean overall quality score using NOS for all included studies is 7.11 with a standard deviation of 1.46. We found that 70%, 59% and 32% of included studies obtained maximum scores for each of the 3 subcategories of NOS which are selection, comparability and outcomes, respectively. A histogram of this data is depicted in Fig. 2 and the individual scores are available in Table 3.

**Table 3:** Newcastle-Ottawa Score for included studies

ID	Articles	Representativeness of exposed cohort	Selection of non-exposed cohort	Ascertainment of exposure	Demonstration outcome was not present at start of study	Study controls for important factor	Study controls for additional factors	Assessment of outcome	follow-up duration	Adequacy of follow-up	Overall Quality Score
		Selection				Comparability		Outcome			
1	Adams et al.	*	*	*	*	*	–	*	*	–	7
2	Astrom et al.	*	*	*	*	*	–	*	*	–	7
3	Bernstein et al.	*	*	*	*	*	*	*	*	–	8
4	Biskupiak et al.	*	*	*	*	*	–	*	*	–	7
5	Bradford et al.	*	*	–	*	–	*	*	–	–	5
6	Cheung et al.	–	*	*	*	–	–	*	*	–	5
7	Coplan et al.	*	*	*	*	–	–	*	–	–	5
8	Dhawale et al.	–	*	*	*	–	–	*	*	*	6
9	Elmelund et al.	*	*	*	*	*	*	*	*	*	9
10	Fattah et al.	*	*	*	*	–	–	*	*	*	7
11	Fatti et al.	–	*	*	*	*	*	*	*	*	8
12	Flack et al.	–	*	*	*	*	–	*	*	–	6
13	Fong et al.	*	*	*	*	*	*	*	*	*	9
14	Gao et al.	*	*	*	*	*	–	*	*	*	8
15	Ghate et al.	*	*	*	*	*	*	*	–	–	7
16	Gofman et al.	*	*	*	*	*	*	*	*	*	9
17	Guelinckx et al.	–	*	*	–	–	–	*	*	–	4
18	Haas et al.	*	*	*	*	–	*	*	*	–	7
19	Heintzelman et al.	*	*	*	*	*	*	–	*	*	8
20	Henes et al.	–	*	–	*	–	–	–	*	–	3
21	Jehi et al.	*	*	–	*	*	*	–	–	–	5
22	Kharbanda et al.	*	*	*	*	*	*	*	*	–	8
23	Lasko et al.	*	*	*	*	*	–	*	*	–	7
24	Maahs et al.	*	*	*	*	*	*	*	*	–	8
25	Mahmud et al.	*	*	*	*	*	*	–	*	–	7
26	Mancevski et al.	*	*	*	*	*	*	*	*	*	9
27	McCoy et al.	*	*	*	*	*	*	*	*	*	9
28	Nannetti et al.	*	*	*	*	–	–	*	*	–	6
29	Pan et al.	*	*	*	*	*	*	*	*	*	9
30	Patterson et al.	*	*	*	*	*	*	*	*	*	9
31	Pirraglia et al.	*	*	*	*	*	*	*	*	–	8
32	Roth et al.	*	*	*	–	*	*	*	*	*	8
33	Ruiz et al.	*	*	*	*	*	*	*	*	–	8
34	Sarafoglou et al.	*	*	*	*	*	*	*	*	*	9
35	Schwartz et al.	*	*	*	*	*	*	*	*	–	8
36	Snijder et al.	*	*	–	*	*	*	*	*	–	7
37	Sy et al.	*	*	*	*	–	–	*	*	–	6
38	Tamayo et al.	*	*	*	*	*	*	*	*	–	8
39	Tanabe et al.	*	*	*	*	*	–	–	*	–	6
40	Ting et al.	–	*	*	–	*	*	*	*	*	7
41	Ullrich et al.	–	*	*	*	*	*	–	*	–	6
42	Walker et al.	*	*	*	–	*	*	–	*	–	6
43	Wong et al.	*	*	*	*	*	*	*	*	–	8
44	Zechmann et al.	*	*	*	*	–	–	*	*	–	6

## Discussion

We conducted a systematic review of articles that used longitudinal data collected as part of patients’ usual care. We found that reporting of variability in number or timing of visits was suboptimal, and reporting on the potential informativeness of visit times was rare. Furthermore, a method specifically designed to account for informativeness of visit times was used in just one of the 44 studies. On using the NOS scale to assess study quality, only 14 studies (32%) reported adequate cohort follow-up.

When visit times are irregular, it is important the investigate whether visit times are informative, that is, whether visit and outcome processes are dependent [2, 5]. This should also be reported on, so that the reader is aware of the scope for bias due to visit irregularity; this is very similar to the need to investigate and report missingness mechanisms when missing data is present [28, 29]. Only one study detailed analysis in the methods section designed to check for informativeness of the visit times, while in a further five studies informativeness was inferred by the reviewers but neither named as a potential source of bias nor accounted for in the analysis.

Our findings are consistent with an overall context of poor reporting. For example, a recent systematic review of studies using routinely collected health data found that reporting was poor, with 30% reporting study design in the title or abstract, and only 41% providing sufficient information to formulate a research question [30]. In the context of longitudinal prognostic studies in lupus, a systematic review found that 56% of studies had a high risk of bias with regards to attrition [31]. Only 43% of prospective cohort studies were found to have reported the amount of missing data [32], and only half of trials with missing longitudinal data explained the reasons for their choice of missing data method [33]. Given that this occurs despite considerable efforts to improve the reporting of observational studies and missing data (including the widely endorsed STROBE reporting guideline [28]), it is not surprising that few studies report on the degree and informativeness of irregular visits, for which there is no guidance in the literature.

Poor reporting makes it impossible to determine definitively whether lack of use of methods for longitudinal data with irregular follow-up is due to lack of need. However, the inclusion/exclusion criteria were designed to capture studies with irregular follow-up, and for such studies the set of circumstances under which a simple GEE or linear mixed model leads to unbiased inferences is extremely narrow. For a GEE this requires visit times to be independent of both past and future outcomes. This is generally implausible when data is collected as part of usual care, since usually patients will be seen more often when unwell. A linear mixed effects model yields unbiased estimates of regression coefficients in the presence of informative visit times only if the predictors of visit times are included in the mixed model [4]. Moreover, in the case of repeated measures analysis the outcome should not be dependent on time if the timings of the visits vary. Some studies attempt to standardize the number of data points per patient used in regression models, e.g. by taking the mean measurement per patient per year. While this is effective at ensuring that each patient is equally represented, it overlooks the fact that certain types of measurement are likely over-represented. For example, if patients visit more often when unwell, then the mean of the observed measurements in any given year over-estimates the patient’s burden of disease for that year. We thus hypothesize that among the 44 studies identified, many did in fact need analytic techniques specifically designed to account for an informative visit process.

In each of the five papers that identified predictors of both visit times and outcomes but that did not use a method to account for the informative visit process, an inverse intensity weighted analysis was feasible. Such analyses could be made more accessible through availability of suitable software. Inverse intensity weighted GEEs can be fitted using PROC GENMOD in SAS or geeglm in R after calculating the intensity separately, but a one-step estimation function would be preferable. Similarly, there is no R package or set of SAS macros for fitting semi-parametric joint models.

While a 2015 Web of Science citation analysis suggested that methods that account for informative visit times had been used just three times in the medical literature, this review identified a fourth [22]. This paper was not identified by the citation analysis as the reference to the inverse-intensity weighting method was incorrect (first and last author names were reversed).

The analysis of longitudinal data subject to irregular follow-up has been an active area of research in the past decade [2, 6, 7, 34, 35]. However, our findings suggest that knowledge of these methods has yet to be translated into medical research. These methods have received less attention than those used in handling missing data [34]. The uptake of biostatistical methods in medical research is facilitated through collaboration and the availability of software to implement these methods [36]. A proactive approach is needed to bridge the knowledge gap with respect to longitudinal data subject to irregular follow-up. There is also a need for standards for reporting longitudinal studies subject to irregular follow-up, both in terms of the extent of irregularity and its informativeness. Improving the quality of reporting and using methods that account for the informative nature of the visit process will reduce the risk of bias and hence improve the quality of evidence in the medical literature.

## Recommendations

The best way to avoid bias due to irregular observation is through study design. In a prospective study this can be accomplished by specifying visit times a priori. Some studies, however, follow clinic-based cohorts where visits are on an as-needed basis and vary among patients; adding additional study visits would substantially increase the cost of the study. Likewise, in a retrospective study the visit times are already set. In these cases, analysis should begin with an investigation of the variability of visit times, and by looking at whether there are any factors that predict visit frequency. The former can be accomplished by descriptive statistics on numbers of visits and gaps between visits, and the latter by a recurrent event analysis on the visit times. If important predictors of visit frequency are found, a method that accounts for the informativeness of visit times should be used. Such methods include inverse intensity weighting [2, 8–10] and semi-parametric joint models [11–14]. See Pullenayegum & Lim [5] for a review together with guidance on when to use each method.

## Conclusion

We found a low proportion of studies reporting on the potential informativeness of visit times. There is a need for guidance to researchers on the potential for bias and the reporting of longitudinal studies subject to irregular follow-up.

## Additional file

Additional file 1: Search strategy and the list of eligible articles. (DOCX 21 kb)

## Abbreviations

BMI: Body mass index
DBS: Deep-brain stimulation
GGE: Generalized Estimating Eqs.
HDL: High-density lipoprotein
IIW: Inverse-intensity weighing
