# Supplementary material for: Longitudinal studies that use data collected as part of usual care risk reporting biased results: a systematic review
Source: BMC Med Res Methodol. 2017 Sep 6;17:133. doi: 10.1186/s12874-017-0418-1 (PMC5588621; doi:10.1186/s12874-017-0418-1)
Supplement: Additional file 1: — Search strategy and the list of eligible articles. (DOCX 21 kb) [file 12874_2017_418_MOESM1_ESM.docx]

**Additional file**

Appendix A: Search Strategy

1.(regression OR regress OR regressed OR repeated measures OR generalized estimating equation OR gee OR mixed model OR random effect).ab.

2.(longitudinal OR repeated measures OR generalized estimating equation OR gee OR mixed model OR random effect OR panel OR retrospective cohort OR prospective cohort).ab.

3.(administrative data OR chart OR medical record OR hospital record OR routine data).ab.

4.1 and 2 and 3

5.limit 4 to yr=”2005-Current”

6.(administrative data OR chart OR medical record OR hospital record OR routine data).ab.

7.(administrative data OR chart OR medical record OR hospital record OR routine data).ti.

8.(administrative data OR chart OR medical record OR hospital record OR routine data).kw.

9.(administrative data OR chart OR medical record OR hospital record OR routine data).kf.

10.(administrative data OR chart OR medical record OR hospital record OR routine data).ot.

11.(administrative data OR chart OR medical record OR hospital record OR routine data).ot.

12.(administrative data OR chart OR medical record OR hospital record OR routine data).hw.

13. 6 or 7 or 8 or 9 or 10 or 11 or 12

14. (administrative data OR chart OR medical record OR hospital record OR routine data).tw.

15. 13 or 14

16. (administrative data OR chart OR medical record OR hospital record OR routine data).fs.

17. 1 and 2 and 15

18. limit 17 to yr=”2005-2015”

Appendix B: List of Eligible Articles

| 1. Adams, A.S., et al. Medication adherence and racial differences in A1C control. Diabetes Care 31, 916-921 (2008). |
| --- |
| 1. Astrom, S., Stenlund, H. & Linden, C. Intraocular pressure changes over 21 years - a longitudinal age-cohort study in northern Sweden. Acta Ophthalmol 92, 417-420 (2014). |
| 1. Bernstein, I.M., Thibault, A., Mongeon, J.A. & Badger, G.J. The influence of pregnancy on arterial compliance. Obstetrics & Gynecology 105, 621-625 (2005). |
| 1. Biskupiak, J.E., Kim, J., Phatak, H. & Wu, D. Prevalence of high-risk cardiovascular conditions and the status of hypertension management among hypertensive adults 65 years and older in the United States: analysis of a primary care electronic medical records database. Journal of Clinical Hypertension 12, 935-944 (2010). |
| 1. Bradford, W.D., Kleit, A.N., Nietert, P.J. & Ornstein, S. Effects of direct-to-consumer advertising of hydroxymethylglutaryl coenzyme a reductase inhibitors on attainment of LDL-C goals. Clinical Therapeutics 28, 2105-2118; discussion 2104 (2006). |
| 1. Cheung, T., et al. Longitudinal impedance variability in patients with chronically implanted DBS devices. Brain Stimul 6, 746-751 (2013). |
| 1. Coplan, J. & Jawad, A.F. Modeling clinical outcome of children with autistic spectrum disorders. Pediatrics 116, 117-122 (2005). |
| 1. Dhawale, A.A., et al. Casting for infantile scoliosis: the pitfall of increased peak inspiratory pressure. J Pediatr Orthop 33, 63-67 (2013). |
| 1. Elmelund, M., Oturai, P.S. & Biering-Sorensen, F. 50 years follow-up on plasma creatinine levels after spinal cord injury. Spinal Cord 52, 368-372 (2014). |
| 1. Fattah, A.Y., et al. Cephalometric Outcomes of Orthognathic Surgery in Hemifacial Microsomia. J Craniofac Surg (2014). |
| 1. Fatti, G., Bock, P., Grimwood, A. & Eley, B. Increased vulnerability of rural children on antiretroviral therapy attending public health facilities in South Africa: a retrospective cohort study. Journal of the International AIDS Society 13, 46 (2010). |
| 1. Flack, J.M., et al. Influence of albuminuria and glomerular filtration rate on blood pressure response to antihypertensive drug therapy. Vasc Health Risk Manag 3, 1029-1037 (2007). |
| 1. Fong, T.G., et al. Delirium accelerates cognitive decline in Alzheimer disease. Neurology 72, 1570-1575 (2009). |
| 1. Gao, S., et al. Redefined blood pressure variability measure and its association with mortality in elderly primary care patients. Hypertension 64, 45-52 (2014). |
| 1. Ghate, S.R., et al. Association between second-generation antipsychotics and changes in body mass index in adolescents. Journal of Adolescent Health 52, 336-343 (2013). |
| 1. Gofman, I. & Ducore, J. Risk factors for the development of obesity in children surviving ALL and NHL. J Pediatr Hematol Oncol 31, 101-107 (2009). |
| 1. Guelinckx, I., Beckers, K., Vansant, G. & Devlieger, R. Construction of weight gain charts in a low-risk obstetric Belgian population. Gynecol Obstet Invest 69, 57-61 (2010). |
| 1. Haas, W.C., Moore, J.B., Kaplan, M. & Lazorick, S. Outcomes from a medical weight loss program: primary care clinics versus weight loss clinics. American Journal of Medicine 125, 603.e607-611 (2012). |
| 1. Heintzelman, N.H., et al. Longitudinal analysis of pain in patients with metastatic prostate cancer using natural language processing of medical record text. Journal of the American Medical Informatics Association 20, 898-905 (2013). |
| 1. Henes, S.T., Collier, D.N., Morrissey, S.L., Cummings, D.M. & Kolasa, K.M. Medical nutrition therapy for overweight youth in their medical home: the KIDPOWER experience. Patient Education & Counseling 81, 43-46 (2010). |
| 1. Jehi, L., Tesar, G., Obuchowski, N., Novak, E. & Najm, I. Quality of life in 1931 adult patients with epilepsy: seizures do not tell the whole story. Epilepsy Behav 22, 723-727 (2011). |
| 1. Kharbanda, E.O., et al. Initiation of oral contraceptives and changes in blood pressure and body mass index in healthy adolescents. J Pediatr 165, 1029-1033 (2014). |
| 1. Lasko, T.A., Denny, J.C. & Levy, M.A. Computational phenotype discovery using unsupervised feature learning over noisy, sparse, and irregular clinical data. PloS one 8, e66341 (2013). |
| 1. Maahs, D.M., et al. Longitudinal lipid screening and use of lipid-lowering medications in pediatric type 1 diabetes. J Pediatr 150, 146-150, 150 e141-142 (2007). |
| 1. Mahmud, S., Lou, W.W. & Johnston, N.W. A probit- log- skew-normal mixture model for repeated measures data with excess zeros, with application to a cohort study of paediatric respiratory symptoms. BMC medical research methodology 10, 55 (2010). |
| 1. Mancevski, B., et al. Lifelong course of positive and negative symptoms in chronically institutionalized patients with schizophrenia. Psychopathology 40, 83-92 (2007). |
| 1. McCoy, A.A., Fox, M.A., Schaubel, D.E. & Ayyangar, R.N. Weight gain in children with hypertonia of cerebral origin receiving intrathecal baclofen therapy. Archives of Physical Medicine & Rehabilitation 87, 1503-1508 (2006). |
| 1. Nannetti, L., Paci, M., Baccini, M., Rinaldi, L.A. & Taiti, P.G. Recovery from stroke in patients with diabetes mellitus. Journal of Diabetes & its Complications 23, 249-254 (2009). |
| 1. Pan, I.J., et al. Lactational exposure to polychlorinated biphenyls, dichlorodiphenyltrichloroethane, and dichlorodiphenyldichloroethylene and infant growth: an analysis of the Pregnancy, Infection, and Nutrition Babies Study. Paediatric and Perinatal Epidemiology 24, 262-271 (2010). |
| 1. Patterson, A.J., et al. Association between white matter ischaemia and carotid plaque morphology as defined by high-resolution in vivo MRI. Eur J Vasc Endovasc Surg 38, 149-154 (2009). |
| 1. Pirraglia, P.A., et al. Benefits of a primary care clinic co-located and integrated in a mental health setting for veterans with serious mental illness. Preventing Chronic Disease 9, E51 (2012). |
| 1. Roth, L.S., Chande, N., Ponich, T., Roth, M.L. & Gregor, J. Predictors of disease severity in ulcerative colitis patients from Southwestern Ontario. World Journal of Gastroenterology 16, 232-236 (2010). |
| 1. Ruiz, A., et al. Exploratory analysis of seven Alzheimer's disease genes: disease progression. Neurobiology of Aging 34, 1310.e1311-1317 (2013). |
| 1. Sarafoglou, K., et al. Impact of hydrocortisone on adult height in congenital adrenal hyperplasia-the Minnesota cohort. J Pediatr 164, 1141-1146 e1141 (2014). |
| 1. Schwartz, B.S., et al. Attention deficit disorder, stimulant use, and childhood body mass index trajectory. Pediatrics 133, 668-676 (2014). |
| 1. Snijder, C.A., et al. Occupational exposure to chemicals and fetal growth: the Generation R Study. Hum Reprod 27, 910-920 (2012). |
| 1. Sy, K., et al. Safety and effectiveness of radiologic percutaneous gastrostomy and gastro jejunostomy in children with cardiac disease. AJR. American Journal of Roentgenology 191, 1169-1174 (2008). |
| 1. Tamayo, C., Manlhiot, C., Patterson, K., Lalani, S. & McCrindle, B.W. Longitudinal evaluation of the prevalence of overweight/obesity in children with congenital heart disease. Can J Cardiol 31, 117-123 (2015). |
| 1. Tanabe, P., Hafner, J.W., Martinovich, Z. & Artz, N. Adult emergency department patients with sickle cell pain crisis: results from a quality improvement learning collaborative model to improve analgesic management. Academic Emergency Medicine 19, 430-438 (2012). |
| 1. Ting, G., et al. Performance of a rheumatoid arthritis records-based index of severity. Journal of Rheumatology 32, 1679-1687 (2005). |
| 1. Ullrich, P.M., et al. Pain, depression, and health care utilization over time after spinal cord injury. Rehabilitation Psychology 58, 158-165 (2013). |
| 1. Walker, M.S., Schwartzberg, L.S., Stepanski, E.J. & Fortner, B.V. A retrospective study of quality of life in a community sample of patients with early stage breast cancer. Breast Cancer Research & Treatment 115, 415-422 (2009). |
| 1. Wong, E.S., et al. BMI trajectories among the severely obese: results from an electronic medical record population. Obesity 20, 2107-2112 (2012).  \| 1. Zechmann, C.M., et al. Changes of prostate gland volume with and without androgen deprivation after intensity modulated radiotherapy - A follow-up study. Radiother Oncol 90, 408-412 (2009). \| \| --- \| |
